# Sustainable Development of African Countries: Minding Public Life, Education, and Welfare

**DOI:** 10.3389/fpubh.2021.748845

**Published:** 2021-11-11

**Authors:** Danyang Li, Guosheng He, Hui Jin, Fu-Sheng Tsai

**Affiliations:** ^1^Department of Finance, School of Economics, Liaoning University, Shenyang, China; ^2^Department of Economics, School of Economics and Management, Zhejiang Sci-Tech University, Hangzhou, China; ^3^North China University of Water Resources and Electric Power, Zhengzhou, China; ^4^Department of Business Administration, Cheng Shiu University, Kaohsiung, Taiwan; ^5^Center for Environmental Toxin and Emerging-Contaminant Research, Cheng Shiu University, Kaohsiung, Taiwan; ^6^Super Micro Mass Research and Technology Center, Cheng Shiu University, Kaohsiung, Taiwan

**Keywords:** sustainable development, education, welfare, Africa, public life

## Abstract

Sustainable development (SD) has increasingly played a key background role in government policymaking across the world, especially for the least developed countries in Africa. Therefore, the purpose of our research is to study the SD of African countries in public life, education, and welfare, and then to help policy makers better monitor the status of sustainable development and formulate development policies in these aspects. We firstly propose a new method to assess the SD in public life, education, and welfare. Then we assess the SD status in 51 African countries as well as other countries in the world. After that, we also make a comparison between African countries and the countries in other continents.

## Introduction

Since the UN 2030 Agenda and Sustainable Development Goals (SDGs) were adopted by all 193 member states, governments and researchers are increasingly trying to monitor performance in sustainability (1). The monitoring of sustainable development (SD) performance has made it necessary to construct a composite index system (2). A composite index, which can evaluate national SD comprehensively and objectively, will not only provide comprehensive status of sustainability, but also provide a policy basis for governments to formulate and implement public policies (3).

As reviewed in the next section, a list of indices have been proposed for sustainability assessment, and it was found that the SD performance of African countries is at the lowest level in the world (4–7). Moreover, most of the existing studies have pointed out improving the SD level of African countries is the key to achieving the global SDGs, and Africa is the continent which needs the most attention (3). The reason is that the leading SD countries have achieved a high SD level, the countries with medium SD level have found a way to increase SD, while the low SD countries (mainly in Africa) are still unable to prosper (1).

At present, many literature papers have studied the SD of African countries, especially in the aspects of governance, economy, resources, and environment, like Selmier and Newenham-Kahindi (8), Mutiiria et al. (9), Asongu and Nnanna (10), and Liyanage et al. (11), but the research on public life, education, and welfare is not enough. For example, Atisa et al. (12) studied legal structures, governance, and sustainable development in African countries.

Therefore, this paper aims to study the SD of African countries in public life, education, and welfare, so as to help governments to monitor the status of sustainability and formulate sustainable development policies in these aspects. We firstly propose a new method for the assessment of SD in public life, education, and welfare based on existing studies. Then we assess and analyze the SD of African countries with these aspects in mind. After that, we also make a comparison between African countries and other countries in the world.

The rest of the paper is organized into five sections. Section Literature Review is the literature review. Section Methods and Data describes the method for the assessment of SD in public life, education, and welfare, as well the data source. Section Results includes the results. Section Discussion: A Comparison Between African and Other Countries compares the SD of African countries and that of other countries. Section Conclusion concludes the findings.

## Literature Review

### The Concept of Sustainable Development

The concept of sustainable development originated from ecology, but with the progress of related studies, it gradually evolved into a comprehensive concept including economy, society, and environment. Sustainable development has become the focus of global attention and controversy, especially after the Our Common Future report from the Brundtland Commission (13). The report defines sustainable development as development that meets the needs of the present generation without compromising the needs of the future generation. It may be considered as the first definition of sustainable development, emphasizing its intergenerational and ecologically oriented aspect (2). Although the concept of sustainable development originated from ecology, it has brought together many disciplines and interests, involving ecology together with environmental, economic, and societal aspects (14, 15). And sustainable development is also considered as a cross-cutting concept which includes three dimensions, namely social, economic, and environment aspects (16). As Guillén-Royo (17) has pointed out, sustainable development demands action on its three dimensions by development policies fostering economic growth, greater social equality, and the reduction of negative environmental impacts. Kwatra et al. (18) put forward a similar concept, sustainable development is a multi-dimensional concept, which emphasizes integration and striking a dynamic balance between economic, social, and environmental aspects in a region to ensure inter-generational and intragenerational equity. In recent years, although sustainable development is defined slightly differently by various researchers, it is the general trend that sustainable development is a concept including three dimensions of economy, society, and environment. As Jin et al. (3) concluded, sustainable development is to coordinate economic, social, and environmental development, so as to balance the intra-generational welfare, and then maximize the total welfare of generations.

### Indices for Sustainable Development Assessment

After the concept of sustainable development was put forward, a growing list of studies were devoted to building a composite index for sustainability assessment. There are many classic and well-known examples, like the Index of Sustainable Economic Welfare (19, 20), ecological footprint (21), genuine savings (22), Environmental Sustainability Index (23), Environmental Performance Index (2006), and so on. In addition, many widely referenced sustainable development indices are constructed by international organizations, such as the UN's Sustainable Development Goals Index (24) and the United Nations Development Program's (25) Human Development Index (HDI).

The Human Development Index (HDI) is the one of the most widely used and referenced indices (26). The HDI is an excellent index, and famous for its simple composition, representative sub-indicators, and rich connotation (7). It consists of three equal-weighted indicators: income, life expectancy, and education. But the HDI is criticized and even suspected of not being a “strict” sustainable development index, because it does not have indicators on environmental and resource dimensions (6, 27).

Therefore, some studies put forward modified indices for the HDI by adding indicators of resource and environment, such as the Human Sustainable Development Index (HSDI) constructed by Bravo (6), Human Green Development Index (HGDI) by Li et al. (5), and the National Sustainable Development Index (NSDI) by Jin et al. (3). The HSDI, HGDI, and NSDI are all regarded as “modified indices” or improved schemes of the HDI, but they are quite different in composition and connotation. Among these modified indices of the HDI, the NSDI is considered as a relatively complete indicator, and more in line with the concept of sustainable development (28).

## Methods and Data

### The Assessment Framework of SD in Public Life, Education, and Welfare

In order to assess sustainable development in public life, education, and welfare, we adopt the social dimension of the National Sustainable Development Index (see Table 1). The NSDI was built with 12 indicators in economic, social, and environmental dimensions based on the concept of sustainable development (3). Sustainable development is to coordinate economic, social, and environment development, and balance the intra-generational welfare, so as to maximize the total welfare of generations (17, 18). In other words, the government should set sustainable development as a comprehensive goal including economic, social, and environmental dimensions (16). So, governments should pursue a relatively high and fair income for citizens, a potential for economic growth, and a reasonable economic structure to improve the welfare of the present generation, in the economic dimension. From the resource and environmental dimension, the climate and air quality not only reflect the living conditions and quality of human beings in the present generation, but also affect that of future generations, while forests, arable land, and energy consumption represent the current resource and environmental conditions, and affect the performance of economic activities. And in the social dimension, governments should not only improve social welfare, but should also consider social fairness and harmony, thus education for the young, medical treatment for the sick, basic sanitation, and drinking water should be guaranteed. Therefore, Jin et al. (3) suggest that the NSDI should contain these factors, namely “economic growth,” “income level,” and “economic structure” in the economic dimension, “climate,” “air quality,” “forest,” “arable land,” and “energy” in the resource and environmental dimension, and “education,” “health,” “drinking water,” and “sanitation facilities” in the social dimension. And they should select the corresponding indicators for each factor, based on the principles of representativeness, comparability, and data availability. So, we choose the social dimension of the NSDI to study the SD in public life, education, and welfare for Africa countries.

**Table 1:** The social dimension of the National Sustainable Development Index.

**Index**	**Dimension**	**Factor**	**Indicator**	**Premise**
National Sustainable Development Index (NSDI)	Social Dimension	Education	Expected years of schooling	+
		Health	Life expectancy index	+
		Drinking water	Population using at least basic drinking-water sources (%)	+
		Sanitation facilities	Population using at least basic sanitation facilities (%)	+

*(1) The descriptions and data source of the four indicators can be found in Table 2. (2) The NSDI includes economic, environmental, and social dimensions, but we just employed the social dimension for this study. (3) Positive indicators are those whose increasing values represent better performance in sustainable development, such as life expectancy*.

### Normalization

Normalization is a necessary step before the four indicators are aggregated into a composite index. There are many kinds of normalization methods, such as “ranking,” “distance to target,” “Z-Score,” and “min-max” (29, 30). We adopt the min-max method for normalization, because it is simple, established, and widely used (6, 31). According to the min-max method, we divide the four indicators into positive indicators and negative indicators (as shown in the last column of Table 1). Positive indicators are those whose increasing values represent better performance in sustainable development, such as life expectancy. Since the four indicators are all positive, the min-max normalization formula for the positive indicator is shown in Equation (1).


(1) $$\begin{array}{l} {\tilde{x}}_{ij}=\frac{X_{ij}-minX_{ij}}{\text{max }X_{ij}-minX_{ij}} \end{array}$$

In the equation above, *X* is the raw data value, min(*X*) is the minimum observed value of the indicator, max(*X*) is the maximum observed value of the indicator, *X*_*ij*_ is the indicator j of country i, and ${\tilde{x}}_{ij}$ is the result of normalization.

### Weighting for Four Indicators

Weighting is another important step to aggregate all indicators into a sustainable development index. There are some popular weighting methods presented in existing studies, such as equal weights, factor analysis, expert weights, and the entropy method (3, 5, 29, 31). But these methods have limitations in varying degrees. For example, equal weights mean that the weights of all indicators are equal, but the importance of different indicators for sustainable development is obviously different. Similarly, the expert weights method also lacks objectivity (5). And the factor analysis can only estimate weights if correlation exists between indicators (31). The entropy method is considered as an objective weighting technique in sustainable development studies (32).

We use the entropy method to weight each indicator. The entropy method is a weighting technique based on the idea of entropy from information theory. Specifically, information is a measure of the order degree and entropy is a measure of the disorder degree in a system; hence, the smaller the entropy of the indicator, the more information provided by the indicator, and the greater its role and weight in the comprehensive evaluation (32, 33). As Zhang et al. (33) have pointed out, the weight measured by the entropy method represents the relative rate of change of the indicator in a composite index system, while the relative level of each indicator should be calculated by the standardized value of its data. Thus, the entropy method is an objective weighting technique that makes weight judgments based on the size of the data information load. It can reduce the influence of human subjectivity on the evaluation results and makes the evaluation results more realistic (32, 34).

According to the principle of the entropy method, we first normalize each index, as shown in Equation (1). Thus, the entropy value *e*_*j*_ of indicator j could be obtained, as shown in Equations (2) and (3).


(2) $$\begin{array}{l} k=1/\text{ln}\left(n\right) \end{array}$$

(3) $$\begin{array}{l} e_{j}=-k\overset{n}{\underset{i=1}{\sum }}{\tilde{x}}_{ij}ln{\tilde{x}}_{ij} \end{array}$$

The information utility value of indicator *j* is calculated, namely *g*_*j*_ in Equation (4).


(4) $$\begin{array}{l} g_{j}=1-e_{j} \end{array}$$

Finally, we can get the weight of indicator *j*, namely ω_*j*_, as shown in Equation (5).


(5) $$\begin{array}{l} \omega_{j}=g_{j}/\overset{p}{\underset{j=1}{\sum }}g_{j} \end{array}$$

### Data Source and Imputation

We chose to assess the SD in public life, education, and welfare for 179 countries in 2015 (the list of countries is shown in **Table 4**), and then focus on the 51 African countries. These countries were selected by two criteria: (1) all countries had published data of all four indicators (see Table 2); (2) internationally recognized non-sovereign entities were not selected, such as Hong Kong, China. In general, the 179 selected samples included most countries, covering more than 90% of the population and land in the world.

**Table 2:** The descriptions and data sources of the four indicators in the social dimension of the NSDI.

**Indicator**	**Description**	**Source**
Expected years of schooling	Expected years of education (unit: years). Number of years of schooling that a child of school entrance age can expect to receive if prevailing patterns of age-specific enrolment rates persist throughout the child's life.	UNDP
Life expectancy index	According to Atkinson (1970), calculating the life expectancy index can reflect fairness and equality, in the case of unequal distribution factors, based on the data of the UN life table: the higher the index value, the better the health status of residents, and the more equal and fairer the access to health for residents.	UNDP
Population using at least basic drinking-water sources (%)	A population that drinks water from an improved source, provided collection time is not more than 30 min for a round trip. This indicator encompasses people using basic drinking-water services as well as those using safely managed drinking-water services. Improved water sources include piped water, boreholes or tube wells, protected dug wells, protected springs, and packaged or delivered water.	WHO
Population using at least basic sanitation facilities (%)	Percentage of the population using at least basic sanitation services, that is, improved sanitation facilities that are not shared with other households. This indicator encompasses people using basic sanitation services as well as those using safely managed sanitation services. Improved sanitation facilities include flush/pour flush toilets connected to piped sewer systems, septic tanks or pit latrines, pit latrines with slabs (including ventilated pit latrines), and composting toilets.	WHO

*UNDP and WHO are short for the United Nations Development Program and the World Health Organization, respectively*.

Due to the missing data of some indicators in some countries, this paper adopts a different imputation method to fill missing data. The current studies prefer to adopt the imputation method to fill missing data rather than missing out information. This notion is also in tune with works by Campagnolo et al. (35). This paper adopts different imputation methods following the actual situation. Firstly, we use the mean value interpolation method. For example, if the data of 2014 and 2016 are available, but the data of 2015 are missing, we use the average value of 2014 and 2016 to replace the value of 2015. Secondly, we use the nearest neighbor interpolation method. This method is used to dealing with missing data for the variables that are very stable over time. These imputations in instances can distort the results but losing out on data might prove costly to some countries (31).

## Results

This paper measures the weight of four indicators with the entropy method (see the last column of Table 3). As a result, the weights of “education,” “health,” “drinking water,” and “sanitation facilities,” respectively accounted for 36.36, 35.09, 14.60, and 13.95%. It means that education is the most important factor for sustainable development in public life, education, and welfare. And education is as important as health.

**Table 3:** The weights of the four indicators.

**Index**	**Dimension**	**Factor**	**Indicator**	**Weights**
National Sustainable Development Index (NSDI)	Social Dimension	Education	Expected years of schooling	36.36%
		Health	Life expectancy index	35.09%
		Drinking water	Population using at least basic drinking-water sources (%)	14.60%
		Sanitation facilities	Population using at least basic sanitation facilities (%)	13.95%

According to the weights in Table 3, we aggregate the four indicators into a composite index, and assess the SD score for 51 Africa countries as well the other countries (see Table 4 and Appendix Table A1). As a result, the SD score of each country is ranged from 0 to 1. The SD score of each Africa country is shown in Table 4, the top five countries are Mauritius (0.6514), Gabon (0.5836), Gambia (0.5648), Morocco (0.5496), and Togo (0.5474), while the bottom five countries are Mali (0.3863), Mauritania (0.3857), Central African Republic (0.3377), Chad (0.3260), and Niger (0.3031).

**Table 4:** The score of SD in public life, education, and welfare in Africa.

**Country**	**Score**	**Rank**	**Income level**	**Country**	**Score**	**Rank**	**Income level**
Mauritius	0.6514	1	Upper-middle	Congo	0.4795	27	Lower-middle
Gabon	0.5836	2	Upper-middle	Zimbabwe	0.4726	28	Lower-middle
Gambia	0.5648	3	Low	Uganda	0.4701	29	Low
Morocco	0.5496	4	Lower-middle	Kenya	0.4655	30	Lower-middle
Togo	0.5474	5	Low	Angola	0.4624	31	Lower-middle
Seychelles	0.5454	6	High	Cameroon	0.4574	32	Lower-middle
Senegal	0.5378	7	Lower-middle	Guinea	0.4484	33	Low
Malawi	0.5375	8	Low	Egypt	0.4435	34	Lower-middle
Tunisia	0.5366	9	Lower-middle	Burkina Faso	0.4412	35	Low
Cabo Verde	0.5325	10	Lower-middle	Lesotho	0.4348	36	Lower-middle
Eswatini	0.5294	11	Lower-middle	Congo (Dem. Rep.)	0.4270	37	Low
Comoros	0.5240	12	Lower-middle	Liberia	0.4252	38	Low
South Africa	0.5194	13	Upper-middle	Ethiopia	0.4248	39	Low
Botswana	0.5122	14	Upper-middle	Equatorial Guinea	0.4181	40	Upper-middle
Sao Tome and Principe	0.5109	15	Lower-middle	Mozambique	0.4169	41	Low
Zambia	0.5043	16	Lower-middle	South Sudan	0.4046	42	Low
Tanzania	0.5026	17	Lower-middle	Madagascar	0.4002	43	Low
Ghana	0.5023	18	Lower-middle	Djibouti	0.3991	44	Lower-middle
Namibia	0.5009	19	Upper-middle	Eritrea	0.3959	45	Low
Algeria	0.4971	20	Lower-middle	Sierra Leone	0.3952	46	Low
Nigeria	0.4959	21	Lower-middle	Mali	0.3863	47	Low
Burundi	0.4955	22	Low	Mauritania	0.3857	48	Lower-middle
Libya	0.4934	23	Upper-middle	Central African Republic	0.3377	49	Low
Rwanda	0.4904	24	Low	Chad	0.3260	50	Low
Benin	0.4862	25	Lower-middle	Niger	0.3031	51	Lower-middle
Guinea-Bissau	0.4842	26	Low				

*The income level is given by the World Bank*.

The SD score of each country showed distinct characteristics in income level. These countries are divided into four categories according to income levels following the World Bank's standard, namely high, upper-middle, lower-middle, and low income countries. As Table 4 shows, countries with higher SD score tended to be have a higher income level. For example, there are only three low-income countries in the top 20, while 13 low-income countries are in the bottom 20. This means that there may be a positive correlation between income level and SD score. The main reasons are: (1) Those low-income countries have very limited fiscal revenue, leading to insufficient supply of public goods, such as education, medical care, public health, etc. (36); (2) Some low-income countries lack a systematic and efficient public management system, which makes the supply of public goods inefficient (28).

**Figure 1:**
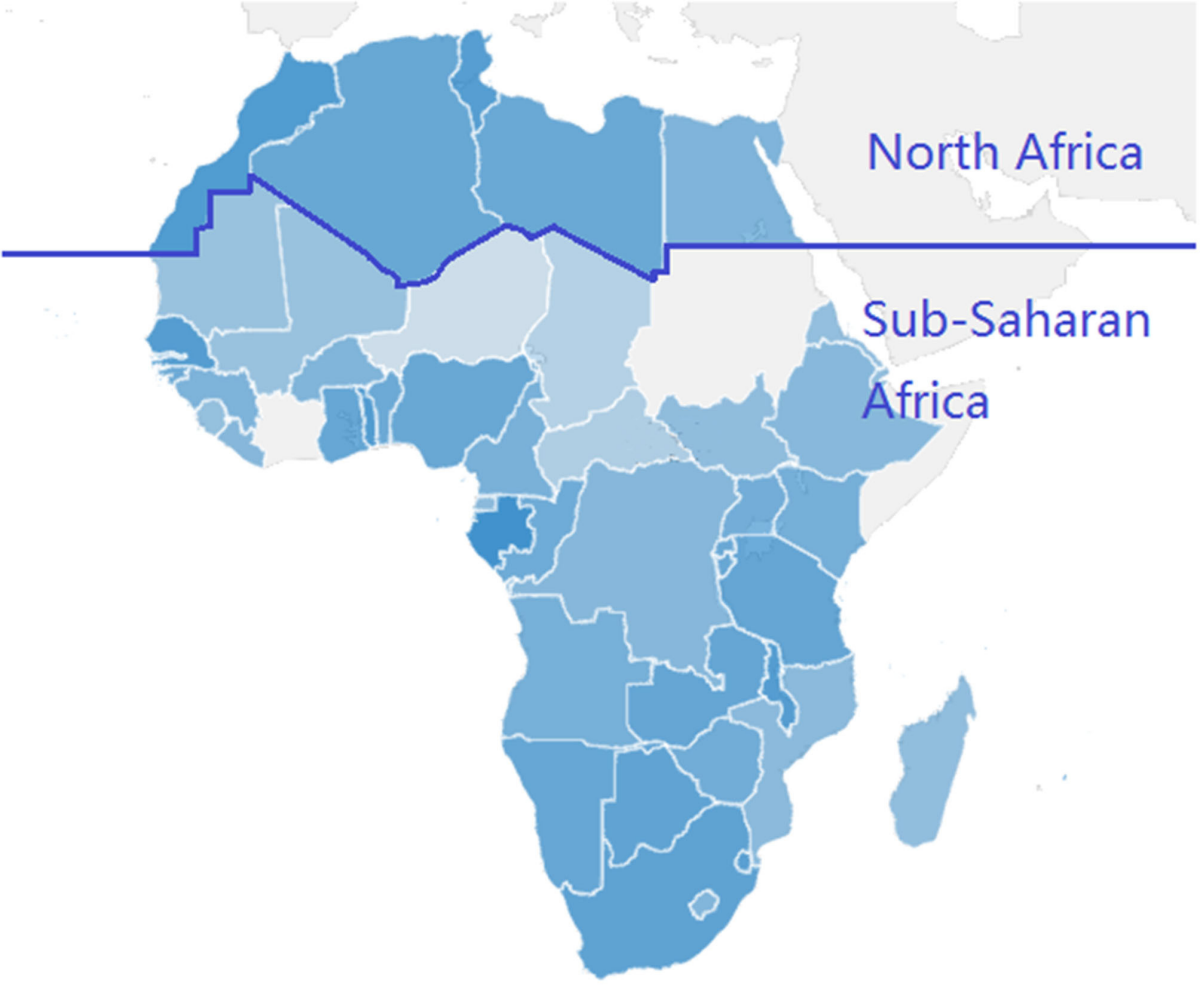
Geographical distribution of SD score in Africa. (1) The darker the blue, the higher the SD score of the country, while gray indicates missing data. (2) Above the dark blue dividing line are North African countries, and below are Sub-Saharan African countries.

Figure 1 shows the geographical distribution of SD score in Africa. It should be noted that the darker the blue, the higher the SD score and SD performance in public life, education, and welfare. The countries in North and South Africa have the deepest blue and the highest SD score, such as South Africa and Morocco. On the contrary, central African countries north of the equator have the lightest blue and the lowest SD score, which means that SD performance is at the bottom level, such as in Central African Republic, Chad, and Niger. In sum, the geographical distribution of SD score shows that SD is high in South and North Africa, while low in the middle.

In addition, we can find the different characteristics of SD status between North African and Sub-Saharan African countries. As Figure 1 shows, above the dark blue dividing line are North African countries, and below are Sub-Saharan African countries. First, the SD performance of North African countries is obviously better than that of Sub-Saharan African countries. This is not only because it is adjacent to the Mediterranean and the climate environment is conducive to survival and development, but also because it is close to those European countries with prosperous economy and society. Second, among Sub-Saharan African countries, the SD score increases from north to south. One of the important reasons is geographical location and climatic environment. And another more important reason is that the countries in the south have established a relatively mature political and institutional system, especially in South Africa.

## Discussion: A Comparison Between African and Other Countries

**Figure 2:**
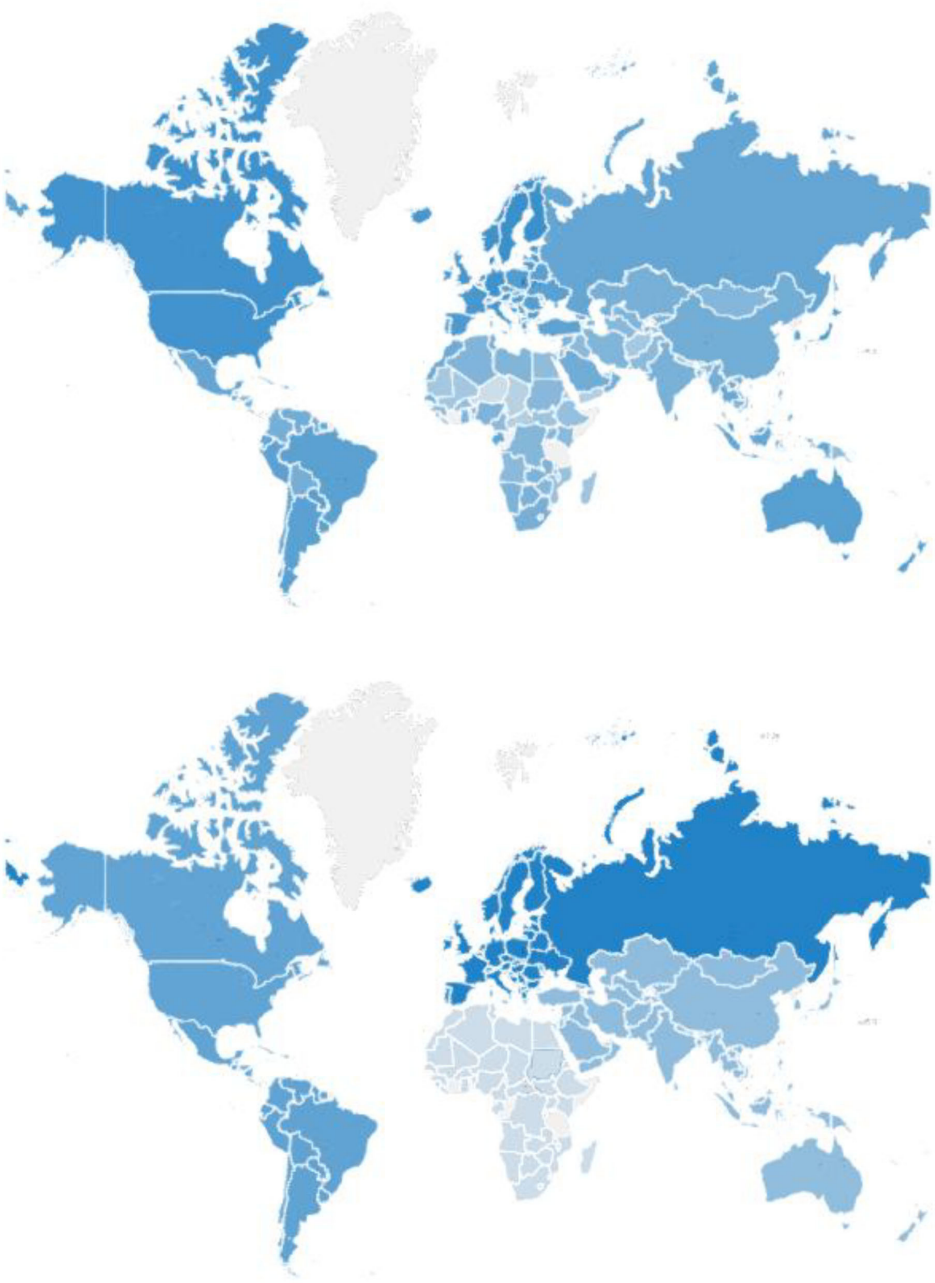
SD score for each country (upper) and continent (lower). The darker the blue, the higher the mean SD score of the country (upper) or the continent (lower).

The SD scores of 179 countries are shown in Figure 2 and Appendix Table A1. As a result, the top 10 countries are Denmark (0.7840), the Netherlands (0.7423), Sweden (0.7095), Finland (0.7075), Norway (0.6960), Germany (0.6915), Canada (0.6895), the United States (0.6856), Belgium (0.6807), and Austria (0.6799), while the bottom 10 countries are Nepal (0.3980), Eritrea (0.3959), Sierra Leone (0.3952), Mali (0.3863), Mauritania (0.3857), Afghanistan (0.3729), Yemen (0.3391), Central African (0.3377), Chad (0.3260), and Niger (0.3031).

The SD score and ranking of each country show distinct characteristics. Most of the high-SD countries are in Europe and North America. The countries with a low SD score are mainly in Africa and Asia. In addition, we find that all the developed countries^1^ are high SD score countries, and most of them are ranked in the top 30, while most of the bottom 30 countries are developing countries in Africa.

There are three main reasons for the poor SD performance in developing countries. First, the level of economy and residents' income is relatively low. Second, the supply of public goods and services is insufficient and inefficient, like education, public health, and environmental protection, due to poor governments or inadequate fiscal revenue (36). Lastly, some developing countries, such as China, are bombarded with such problems as inadequate management and technology of pollution control and resource utilization, while still promoting economic growth at all costs, which damages national sustainable development (3).

The geographical distribution of the SD score is shown in Figure 2. As the figure shows, the darker the blue, the higher the NSDI of the country and the better its performance in sustainable development, while the white indicates missing data. We find that European and North American countries have the highest average SD scores, Africa the lowest, and South America and Asia in the middle.

There is an important reason for that geographical distribution. On the one hand, the countries with a higher economic level always maintain a good performance in sustainable development, because of their established and sound system in public management. On the other hand, those low-income countries not only have a poor economic foundation, but also do not have the above conditions, so they always find it difficult to improve SD performance. Some countries have even been mired in war and extreme poverty.

## Conclusion

This paper aims to study the SD of African countries in public life, education, and welfare, so as to help policy makers better monitor the status of sustainable development and formulate development policies. So, we firstly proposed a new method for the assessment of SD in public life, education, and welfare, and then assessed and analyzed the SD of African countries in these aspects. We found that: (1) there was a positive correlation between income level and SD across African countries; (2) most SD leading countries were in South and North Africa, while most low SD countries were in the middle; and (3) there were different characteristics of SD status between North African and Sub-Saharan African countries.

There is an important research question that needs to be discussed: how to improve the sustainable development level of those low-SD African countries and narrow the development gap among countries? Especially for the Sub-Saharan countries with poor performance in public life, education, and welfare. The cases of North African countries and South Africa may be a reference for those Sub-Saharan countries. Except for natural endowments such as climate, environment, and geographical location, many aspects of North African countries are worthy of reference. First, for those countries still in political chaos, the establishment of a stable political power is the basis of all development. Second, following the practices of European and other developed countries, establishing and improving the judicial, economic, and fiscal institutional systems in combination with the characteristics and development situation of their own countries is vital. After that, taking economic growth as the first priority of national development, and establishing economic and trade cooperation with Europe, the United States, China, and other more developed countries is needed. Lastly, when the economic and income level reaches a certain stage, the government should pay more attention to sustainable development in public life, education, and welfare.

## Data Availability Statement

The original contributions presented in the study are included in the article/Supplementary Material, further inquiries can be directed to the corresponding authors.

## Author Contributions

DL conceived and designed the research, provided guidance throughout the entire research process, and responsible for all R&R works. DL and GH wrote and supplemented the English paper. HJ participated in data analysis. F-ST reviewed and edited the paper. All authors contributed to the article and approved the submitted version.

## Funding

The authors acknowledge funding support from the Major Program Project of the National Social Science Fund of China (No: 19ZDA055), Zhejiang Sci-Tech University (ZSTU) Scientific Research Fund (No: 21092117-Y), and the Prosperity Plan of Philosophy and Social Science Research of ZSTU (No: 21096075-Y).

## Conflict of Interest

The authors declare that the research was conducted in the absence of any commercial or financial relationships that could be construed as a potential conflict of interest.

## Publisher's Note

All claims expressed in this article are solely those of the authors and do not necessarily represent those of their affiliated organizations, or those of the publisher, the editors and the reviewers. Any product that may be evaluated in this article, or claim that may be made by its manufacturer, is not guaranteed or endorsed by the publisher.

## Footnotes

^1^According to the standards of the CIA's World Fact Book and IMF.

## Appendix

**Table A1:** The score of SD in public life, education, and welfare.

**Country**	**Score**	**Rank**	**C**	**Country**	**Score**	**Rank**	**C**
Denmark	0.7840	1	EU	Moldova (Rep.)	0.5351	91	EU
Netherlands	0.7423	2	EU	China	0.5342	92	AS
Sweden	0.7095	3	EU	Cabo Verde	0.5325	93	AF
Finland	0.7075	4	EU	Oman	0.5321	94	AS
Norway	0.6960	5	EU	Qatar	0.5297	95	AS
Germany	0.6915	6	EU	Eswatini	0.5294	96	AF
Canada	0.6895	7	NA	Bolivia	0.5289	97	SA
United States	0.6856	8	NA	Bahrain	0.5281	98	AS
Belgium	0.6807	9	EU	Viet Nam	0.5275	99	AS
Austria	0.6799	10	EU	Azerbaijan	0.5269	100	AS
Iceland	0.6751	11	EU	Kuwait	0.5266	101	AS
Switzerland	0.6695	12	EU	Myanmar	0.5266	102	AS
Luxembourg	0.6693	13	EU	Colombia	0.5256	103	SA
United Kingdom	0.6672	14	EU	Comoros	0.5240	104	AF
Italy	0.6658	15	EU	Timor-Leste	0.5237	105	AS
France	0.6625	16	EU	Sri Lanka	0.5236	106	AS
Portugal	0.6599	17	EU	Nicaragua	0.5211	107	NA
Malta	0.6577	18	EU	Guyana	0.5201	108	SA
Japan	0.6569	19	AS	Kazakhstan	0.5199	109	AS
Singapore	0.6555	20	AS	United Arab Emirates	0.5198	110	AS
Mauritius	0.6514	21	AF	South Africa	0.5194	111	AF
Latvia	0.6487	22	EU	India	0.5186	112	AS
Spain	0.6466	23	EU	Trinidad and Tobago	0.5182	113	NA
Greece	0.6465	24	EU	Samoa	0.5131	114	OC
Ireland	0.6449	25	EU	Botswana	0.5122	115	AF
Estonia	0.6433	26	EU	Sao Tome and Principe	0.5109	116	AF
North Macedonia	0.6425	27	EU	Kyrgyzstan	0.5098	117	AS
Hungary	0.6418	28	EU	Cuba	0.5094	118	NA
Poland	0.6365	29	EU	Jordan	0.5094	119	AS
Croatia	0.6365	30	EU	Senegal	0.5078	120	AF
Slovenia	0.6358	31	EU	Zambia	0.5043	121	AF
Romania	0.6315	32	EU	Tanzania	0.5026	122	AF
New Zealand	0.6291	33	OC	Ghana	0.5023	123	AF
Panama	0.6289	34	NA	Namibia	0.5009	124	AF
Costa Rica	0.6269	35	NA	Tonga	0.5005	125	OC
Lithuania	0.6267	36	EU	Algeria	0.4971	126	AF
Malaysia	0.6257	37	AS	Nigeria	0.4959	127	AF
Andorra	0.6249	38	EU	Burundi	0.4955	128	AF
Slovakia	0.6240	39	EU	Uzbekistan	0.4941	129	AS
Czechia	0.6227	40	EU	Libya	0.4934	130	AF
Bulgaria	0.6207	41	EU	Syrian Arab Republic	0.4928	131	AS
Australia	0.6206	42	OC	Iran	0.4916	132	AS
Paraguay	0.6202	43	SA	Solomon Islands	0.4905	133	OC
Cyprus	0.6161	44	EU	Rwanda	0.4904	134	AF
Belarus	0.6156	45	EU	Belize	0.4881	135	NA
Albania	0.6145	46	EU	Micronesia	0.4868	136	OC
Bosnia and Herzegovina	0.6135	47	EU	Cambodia	0.4863	137	AS
Israel	0.6126	48	AS	Benin	0.4862	138	AF
Brazil	0.6111	49	SA	Bhutan	0.4856	139	AS
Argentina	0.6102	50	SA	Guinea-Bissau	0.4842	140	AF
Ukraine	0.6097	51	EU	Mongolia	0.4823	141	AS
Uruguay	0.6088	52	SA	Congo	0.4795	142	AF
Brunei Darussalam	0.6064	53	AS	Vanuatu	0.4792	143	OC
Korea (Rep.)	0.6064	54	AS	Bangladesh	0.4745	144	AS
Peru	0.6030	55	SA	Zimbabwe	0.4726	145	AF
Montenegro	0.6030	56	EU	Sudan	0.4710	146	AS
Dominican Republic	0.6028	57	NA	Uganda	0.4701	147	AF
Grenada	0.6012	58	NA	Turkmenistan	0.4698	148	AS
Barbados	0.6011	59	NA	Kiribati	0.4660	149	OC
Turkey	0.6000	60	AS	Haiti	0.4658	150	NA
Chile	0.5951	61	SA	Kenya	0.4655	151	AF
Suriname	0.5898	62	SA	Tajikistan	0.4654	152	AS
Serbia	0.5894	63	EU	Angola	0.4624	153	AF
Bahamas	0.5887	64	NA	Iraq	0.4617	154	AS
Fiji	0.5883	65	OC	Pakistan	0.4588	155	AS
Gabon	0.5836	66	AF	Cameroon	0.4574	156	AF
Mexico	0.5794	67	NA	Guinea	0.4484	157	AF
Russia	0.5786	68	EU	Egypt	0.4435	158	AF
Dominica	0.5773	69	NA	Burkina Faso	0.4412	159	AF
Maldives	0.5736	70	AS	Papua New Guinea	0.4366	160	OC
Indonesia	0.5734	71	AS	Lesotho	0.4348	161	AF
Ecuador	0.5728	72	SA	Congo (Dem. Rep.)	0.4270	162	AF
Jamaica	0.5714	73	NA	Liberia	0.4252	163	AF
Philippines	0.5707	74	AS	Ethiopia	0.4248	164	AF
El Salvador	0.5654	75	NA	Equatorial Guinea	0.4181	165	AF
Gambia	0.5648	76	AF	Mozambique	0.4169	166	AF
Lebanon	0.5646	77	AS	South Sudan	0.4046	167	AF
Thailand	0.5641	78	AS	Madagascar	0.4002	168	AF
Guatemala	0.5637	79	NA	Djibouti	0.3991	169	AF
Georgia	0.5630	80	AS	Nepal	0.3980	170	AS
Honduras	0.5610	81	NA	Eritrea	0.3959	171	AF
Lao	0.5557	82	AS	Sierra Leone	0.3952	172	AF
Morocco	0.5496	83	AF	Mali	0.3863	173	AF
Armenia	0.5487	84	AS	Mauritania	0.3857	174	AF
Togo	0.5474	85	AF	Afghanistan	0.3729	175	AS
Seychelles	0.5454	86	AF	Yemen	0.3391	176	AS
Saudi Arabia	0.5394	87	AS	Central African (Rep.)	0.3377	177	AF
Venezuela	0.5385	88	SA	Chad	0.3260	178	AF
Malawi	0.5375	89	AF	Niger	0.3031	179	AF
Tunisia	0.5366	90	AF				

*C refers to the continent, AS is Asia, AF is Africa, EU is Europe, NA is North America, SA is South America, and OC is Oceania*.

## References

[1] Hametner M Kostetckaia M. Frontrunners and laggards: how fast are the EU member states progressing towards the sustainable development goals? Ecol Econ. (2020) 177:106775. 10.1016/j.ecolecon.2020.106775

[2] Alaimo LS Maggino F. Sustainable development goals indicators at territorial level: conceptual and methodological issues-the Italian perspective. Soc Indic Res. (2020) 147:383–419. 10.1007/s11205-019-02162-4

[3] Jin H Qian X Chin T Zhang H. Global assessment of sustainable development: based on the modification of human development index with entropy method. Sustainability. (2020) 12:1–20. 10.3390/su12083251

[4] Bilbao-Ubillos J. The limits of human development index: the complementary role of economic and social cohesion, development strategies and sustainability. Sustain Dev. (2013) 6:400–12. 10.1002/sd.525

[5] Li XX Liu YM Song T. Calculation of the green development index. Soc Sci China. (2014) 6:69–95.

[6] Bravo G. The human sustainable development index: new calculations and a first critical analysis. Ecol Indic. (2014) 37:145–50. 10.1016/j.ecolind.2013.10.020

[7] Hickel J. The sustainable development index: measuring the ecological efficiency of human development in the Anthropocene. Ecol Econ. (2019) 167:106331. 10.1016/j.ecolecon.2019.05.011

[8] Selmier WT Newenham-Kahindi A. Communities of place, mining multinationals and sustainable development in Africa. J Clean Prod. (2020) 292:125709. 10.1016/j.jclepro.2020.125709

[9] Mutiiria OM Ju Q Dumor K. Sustainable development in Sub-Saharan Africa: the impact of infrastructure on wealth per capita. Int Soc Sci J. (2020) 69:1–14. 10.1111/issj.12226

[10] Asongu S Nnanna J. Inclusive human development in Sub-Saharan Africa. J Enterp Communities. (2020) 14:183–200. 10.1108/JEC-11-2019-011529968214

[11] Liyanage S Netswera FG Motsumi A. Insights from EU policy framework in aligning sustainable finance for sustainable development in Africa and Asia. IJEEP. (2021) 11:459–70. 10.32479/ijeep.9865

[12] Atisa G Zemrani A Weiss M. Decentralized governments: local empowerment and sustainable development challenges in Africa. Environ Dev Sustain. (2021) 23:3349–67. 10.1007/s10668-020-00722-0

[13] World Commission on Environment Development (WCED). Our Common Future. Oxford, UK: University Press (1987).

[14] Ramos TB Caeiro S. Meta-performance evaluation of sustainability indicators. Ecol Indic. (2010) 2:157–66. 10.1016/j.ecolind.2009.04.008

[15] Bolcárová P. KološTa S. Assessment of sustainable development in the EU 27 using aggregated SD index. Ecol Indic. (2015) 48:699–705. 10.1016/j.ecolind.2014.09.001

[16] Goodland R Daly H. Environmental sustainability: universal and non-negotiable. Ecol Appl. (1996) 6:1002. 10.2307/2269583

[17] Guillén-Royo M. Sustainability and wellbeing: Human-scale development in practice. London, UK: Routledges (2016).

[18] Kwatra S Sharma P Kumar A. A critical review of studies related to construction and computation of Sustainable Development Indices. Ecol Indic. (2020) 112:106061. 10.1016/j.ecolind.2019.106061

[19] Cobb CW. The Index for Sustainable Economic Welfare. Boston, USA: Beacon Press (1989).

[20] Cobb CW Cobb JB. The Green National Product: A Proposed Index of Sustainable Economic Welfare. Lanham, MD, USA: University Press of America (1994).

[21] Wackernagel M Rees W. Our Ecological Footprint. Basel, Switzerland: Birkhouse Publishing (1997).

[22] Hamilton K Atkinson G Pearce DW. Genuine Savings as an Indicator of Sustainability. Norwich, UK: CSERGE Working Paper GEC97-03; GSERGE (1997).

[23] Esty DC Levy MA Srebotnjak T Sherbinin D. Environmental Sustainability Index: Benchmarking National Environmental Stewardship. New Haven, CT, USA: Yale Center for Environmental Law Policy (2005).

[24] United Nations (UN). Sustainable Development Report 2019. New York, NY, USA: UN (2019).

[25] United Nations Development Programme (UNDP). Note on Statistics in the Human Development Report. New York, NY, USA: UNDP (2004).

[26] Estoque RC Murayama Y. Social–ecological status index: a preliminary study of its structural composition and application. Ecol Indic. (2014) 43:183–94. 10.1016/j.ecolind.2014.02.031

[27] Ture C. A methodology to analyse the relations of ecological footprint corresponding with human development index: eco-sustainable human development index. Int J Sustain Dev World Ecol. (2013) 1:9–19. 10.1080/13504509.2012.751562

[28] Jin H Jorge Martinez-Vazquez. Sustainable Development and the Optimal Level of Fiscal Expenditure Decentralization. Georgia, USA: ICePP Working Paper Series, #2103, Andrew Young School of Policy Studies, Georgia State University (2021).

[29] Nardo M Saisana M Saltelli A Tarantola S Hoffman A Giovannini E. Handbook on Constructing Composite Indicators: Methodology and User Guide (2005).

[30] Pollesch NL Dale VHN. sustainability assessment: methods and implications. Ecol Econ. (2016) 130:195–208. 10.1016/j.ecolecon.2016.06.018

[31] Khalid AM Sharma S Dubey AK. Data gap analysis, indicator selection and index development: a case for developing economies. Soc Indic Res. (2020) 148:893–960. 10.1007/s11205-019-02225-6

[32] Wang M Zhao X Gong Q Ji Z. Measurement of regional green economy sustainable development ability based on entropy weight-topsis-coupling coordination degree: a case study in Shandong Province, China. Sustainability. (2019) 1:280–95. 10.3390/su11010280

[33] Zhang WM An JW Han C. The application of entropy method in the evaluation of urban sustainable development. J Quant Tech Econ. (2003) 6:115–8.

[34] Ma YM Wu YM Wu BJ. Comprehensive evaluation of sustainable urban development of Yangtze River delta based on entropy method and quadrant method. Econ Geogr. (2015) 6:47–53.

[35] Campagnolo L Eboli F Farnia L Carraro C. Supporting the UN SDGs transition: methodology for sustainability assessment and current worldwide ranking. Economics. (2018) 12:1–31. 10.5018/economics-ejournal.ja.2018-10

[36] Jin H Qian X. How the Chinese government has done with public health from the perspective of the evaluation and comparison about public-health expenditure. Int J Environ Res Public Health. (2020) 17:1–16. 10.3390/ijerph17249272s 33322428 PMC7764182

