# Supplementary material for: Sustainable Development of African Countries: Minding Public Life, Education, and Welfare
Source: Front Public Health. 2021 Nov 11;9:748845. doi: 10.3389/fpubh.2021.748845 (PMC8636036; doi:10.3389/fpubh.2021.748845)
Supplement: Supplementary file 1 [file Data_Sheet_1.PDF]

1. The introduction of Entropy method

Entropy method is a method to calculate the weight of each indicator in the composite indicators system, based on entropy idea of entropy. Specifically, if the data variation degree of an indicator is greater, it means that the greater the information entropy value is, and the greater the role of the indicator in the composite indicators system is, thus the higher weight is (Zhang et al., 2003). According to Zhang et al. (2003), the weight calculated by Entropy method represents the relative rate of change of the indicator in the composite indicators system, while the relative level of each indicator should be figured by the standardized value of its data. The Entropy method's advantage is that it can avoid the shortcoming of subjective evaluation method to some extent (Ma et al., 2015). It implies that Entropy method could make up for the lack of objectivity that HDI and HGDI use subjective evaluation method to calculate the weights of indicators.

According to the introduction of Entropy method above, wo need to relate the different variables having different units with a dimensionless scale from 0 to 1 firstly. As shown in Eq. (1), x_{ij} is the indicator j of country i, and $\tilde{x}_{ij}$ is the result of dimensionless treatment. It should be noted that there are some indicators, like *per capita* CO_2 emissions, reflect the fact that higher values mean a poorer performance of sustainable development, which need to be treated as Eq. (2) shows.

$$\tilde{x}_{ij} = \frac{X_{ij} - minX_{ij}}{maxX_{ij} - minX_{ij}} (1)$$

$$\tilde{x}_{ij} = 1 - \frac{X_{ij} - minX_{ij}}{maxX_{ij} - minX_{ij}} (2)$$

Secondly, calculating the entropy value of each indicator, as shown in Eq. (3) and Eq. (4). e_j is the entropy value of each indicator.

$$k = 1/\ln(n) (3)$$

$$e_j = -k \sum_{i=1}^{n} \tilde{x}_{ij} ln\tilde{x}_{ij} (4)$$

Thirdly, calculating the information utility value of each indicator, namely g_j:

$$g_j = 1 - e_j \quad (5)$$

Finally, the weight of indicator j is obtained, namely ω_j, as shown in Eq. (6).

$$\omega_j = g_j / \sum_{j=1}^{p} g_j (6)$$

2. The code of the Entropy method in Stata 15.0

```
capt prog drop szf
prog szf
set more off
preserve
quietly {
local m = `1'
```

```
local n = `2'
local n2 = `n'+2
if `m'>`n' {
mat A = J(`m',3,.)
}
else {
mat A = J(`n',3,.)
}
forvalues i = 3/`n2' {
local j = `i'-2
capt drop b`j'
ge b`j' = ``i''
su b`j'
ge bss`j' = (b`j'-r(mean))/r(sd)
su bss`j'
replace bss`j' = bss`j'+abs(int(r(min)))+1
su bss`j'
ge bs`j' = bss`j'/r(sum)
}

forvalues j = 1/`n' {
local e`j' = 0
forvalues i = 1/`m' {
local e`j' = `e`j''-1/ln(`m')*bs`j'[`i']*ln(bs`j'[`i'])
}
local d`j' = 1-`e`j''
mat A[`j',2] = `d`j''
}
local d = 0
forvalues p = 1/`n' {
local d = `d'+`d`p''
}
forvalues j = 1/`n' {
local w`j' = `d`j''/`d'
mat A[`j',3] = `w`j''
}
forvalues i = 1/`m' {
local f`i' = 0
forvalues j = 1/`n' {
local f`i' = `f`i''+`w`j''*bs`j'[`i']
}
mat A[`i',1] = `f`i''
}
mat coln A = "fi" "dj" "wj"
```

```
}
matlist A
dis "This program was developed by Jin Hui, Zhejiang University of Technology, Hangzhou, China"
dis "Email: 2111404008@zjut.edu.com"
restore
end
```
