# Supplementary material for: Sustainable Development of African Countries: Minding Public Life, Education, and Welfare
Source: Front Public Health. 2021 Nov 11;9:748845. doi: 10.3389/fpubh.2021.748845 (PMC8636036; doi:10.3389/fpubh.2021.748845)
Supplement: Supplementary file 2 [file Data_Sheet_2.pdf]

Appendix

Table A1. The score of SD in public life, education, and welfare.

Country	Score	Rank	C	Country	Score	Rank	C
Denmark	0.7840	1	EU	Moldova (Rep.)	0.5351	91	EU
Netherlands	0.7423	2	EU	China	0.5342	92	AS
Sweden	0.7095	3	EU	Cabo Verde	0.5325	93	AF
Finland	0.7075	4	EU	Oman	0.5321	94	AS
Norway	0.6960	5	EU	Qatar	0.5297	95	AS
Germany	0.6915	6	EU	Eswatini	0.5294	96	AF
Canada	0.6895	7	NA	Bolivia	0.5289	97	SA
United States	0.6856	8	NA	Bahrain	0.5281	98	AS
Belgium	0.6807	9	EU	Viet Nam	0.5275	99	AS
Austria	0.6799	10	EU	Azerbaijan	0.5269	100	AS
Iceland	0.6751	11	EU	Kuwait	0.5266	101	AS
Switzerland	0.6695	12	EU	Myanmar	0.5266	102	AS
Luxembourg	0.6693	13	EU	Colombia	0.5256	103	SA
United Kingdom	0.6672	14	EU	Comoros	0.5240	104	AF
Italy	0.6658	15	EU	Timor-Leste	0.5237	105	AS
France	0.6625	16	EU	Sri Lanka	0.5236	106	AS
Portugal	0.6599	17	EU	Nicaragua	0.5211	107	NA
Malta	0.6577	18	EU	Guyana	0.5201	108	SA
Japan	0.6569	19	AS	Kazakhstan	0.5199	109	AS
Singapore	0.6555	20	AS	United Arab Emirates	0.5198	110	AS
Mauritius	0.6514	21	AF	South Africa	0.5194	111	AF
Latvia	0.6487	22	EU	India	0.5186	112	AS
Spain	0.6466	23	EU	Trinidad and Tobago	0.5182	113	NA
Greece	0.6465	24	EU	Samoa	0.5131	114	OC
Ireland	0.6449	25	EU	Botswana	0.5122	115	AF
Estonia	0.6433	26	EU	Sao Tome and Principe	0.5109	116	AF
North Macedonia	0.6425	27	EU	Kyrgyzstan	0.5098	117	AS
Hungary	0.6418	28	EU	Cuba	0.5094	118	NA
Poland	0.6365	29	EU	Jordan	0.5094	119	AS
Croatia	0.6365	30	EU	Senegal	0.5078	120	AF
Slovenia	0.6358	31	EU	Zambia	0.5043	121	AF
Romania	0.6315	32	EU	Tanzania	0.5026	122	AF
New Zealand	0.6291	33	OC	Ghana	0.5023	123	AF
Panama	0.6289	34	NA	Namibia	0.5009	124	AF
Costa Rica	0.6269	35	NA	Tonga	0.5005	125	OC
Lithuania	0.6267	36	EU	Algeria	0.4971	126	AF
Malaysia	0.6257	37	AS	Nigeria	0.4959	127	AF
Andorra	0.6249	38	EU	Burundi	0.4955	128	AF
Slovakia	0.6240	39	EU	Uzbekistan	0.4941	129	AS
Czechia	0.6227	40	EU	Libya	0.4934	130	AF
Bulgaria	0.6207	41	EU	Syrian Arab Republic	0.4928	131	AS
Australia	0.6206	42	OC	Iran	0.4916	132	AS
Paraguay	0.6202	43	SA	Solomon Islands	0.4905	133	OC
Cyprus	0.6161	44	EU	Rwanda	0.4904	134	AF
Belarus	0.6156	45	EU	Belize	0.4881	135	NA
Albania	0.6145	46	EU	Micronesia	0.4868	136	OC
Bosnia and Herzegovina	0.6135	47	EU	Cambodia	0.4863	137	AS
Israel	0.6126	48	AS	Benin	0.4862	138	AF
Brazil	0.6111	49	SA	Bhutan	0.4856	139	AS
Argentina	0.6102	50	SA	Guinea-Bissau	0.4842	140	AF
Ukraine	0.6097	51	EU	Mongolia	0.4823	141	AS
Uruguay	0.6088	52	SA	Congo	0.4795	142	AF
Brunei Darussalam	0.6064	53	AS	Vanuatu	0.4792	143	OC
Korea (Rep.)	0.6064	54	AS	Bangladesh	0.4745	144	AS
Peru	0.6030	55	SA	Zimbabwe	0.4726	145	AF
Montenegro	0.6030	56	EU	Sudan	0.4710	146	AS
Dominican Republic	0.6028	57	NA	Uganda	0.4701	147	AF
Grenada	0.6012	58	NA	Turkmenistan	0.4698	148	AS
Barbados	0.6011	59	NA	Kiribati	0.4660	149	OC

Turkey	0.6000	60	AS	Haiti	0.4658	150	NA
Chile	0.5951	61	SA	Kenya	0.4655	151	AF
Suriname	0.5898	62	SA	Tajikistan	0.4654	152	AS
Serbia	0.5894	63	EU	Angola	0.4624	153	AF
Bahamas	0.5887	64	NA	Iraq	0.4617	154	AS
Fiji	0.5883	65	OC	Pakistan	0.4588	155	AS
Gabon	0.5836	66	AF	Cameroon	0.4574	156	AF
Mexico	0.5794	67	NA	Guinea	0.4484	157	AF
Russia	0.5786	68	EU	Egypt	0.4435	158	AF
Dominica	0.5773	69	NA	Burkina Faso	0.4412	159	AF
Maldives	0.5736	70	AS	Papua New Guinea	0.4366	160	OC
Indonesia	0.5734	71	AS	Lesotho	0.4348	161	AF
Ecuador	0.5728	72	SA	Congo (Dem. Rep.)	0.4270	162	AF
Jamaica	0.5714	73	NA	Liberia	0.4252	163	AF
Philippines	0.5707	74	AS	Ethiopia	0.4248	164	AF
El Salvador	0.5654	75	NA	Equatorial Guinea	0.4181	165	AF
Gambia	0.5648	76	AF	Mozambique	0.4169	166	AF
Lebanon	0.5646	77	AS	South Sudan	0.4046	167	AF
Thailand	0.5641	78	AS	Madagascar	0.4002	168	AF
Guatemala	0.5637	79	NA	Djibouti	0.3991	169	AF
Georgia	0.5630	80	AS	Nepal	0.3980	170	AS
Honduras	0.5610	81	NA	Eritrea	0.3959	171	AF
Lao	0.5557	82	AS	Sierra Leone	0.3952	172	AF
Morocco	0.5496	83	AF	Mali	0.3863	173	AF
Armenia	0.5487	84	AS	Mauritania	0.3857	174	AF
Togo	0.5474	85	AF	Afghanistan	0.3729	175	AS
Seychelles	0.5454	86	AF	Yemen	0.3391	176	AS
Saudi Arabia	0.5394	87	AS	Central African (Rep.)	0.3377	177	AF
Venezuela	0.5385	88	SA	Chad	0.3260	178	AF
Malawi	0.5375	89	AF	Niger	0.3031	179	AF
Tunisia	0.5366	90	AF				

Note: C refers to the continent, AS is Asia, AF is Africa, EU is Europe, NA is North America, SA is South America, OC is Oceania.
